# Median preoptic area neurons are required for the cooling and febrile activations of brown adipose tissue thermogenesis in rat

**DOI:** 10.1038/s41598-020-74272-w

**Published:** 2020-10-22

**Authors:** Ellen Paula Santos da Conceição, Shaun F. Morrison, Georgina Cano, Pierfrancesco Chiavetta, Domenico Tupone

**Affiliations:** 1 Department of Neurological Surgery, Oregon Health and Science University, 3181 SW Sam Jackson Park Road, Portland, OR 97239-3098 USA; 2 Department of Neuroscience, University of Pittsburgh, Pittsburgh, PA 15260 USA; 3 Department of Biomedical and Neuromotor Science, University of Bologna, 40126 Bologna, Italy

**Keywords:** Neurophysiology, Neural circuits

## Abstract

Within the central neural circuitry for thermoregulation, the balance between excitatory and inhibitory inputs to the dorsomedial hypothalamus (DMH) determines the level of activation of brown adipose tissue (BAT) thermogenesis. We employed neuroanatomical and in vivo electrophysiological techniques to identify a source of excitation to thermogenesis-promoting neurons in the DMH that is required for cold defense and fever. Inhibition of median preoptic area (MnPO) neurons blocked the BAT thermogenic responses during both PGE_2_-induced fever and cold exposure. Disinhibition or direct activation of MnPO neurons induced a BAT thermogenic response in warm rats. Blockade of ionotropic glutamate receptors in the DMH, or brain transection rostral to DMH, blocked cold-evoked or NMDA in MnPO-evoked BAT thermogenesis. RNAscope technique identified a glutamatergic population of MnPO neurons that projects to the DMH and expresses c-Fos following cold exposure. These discoveries relative to the glutamatergic drive to BAT sympathoexcitatory neurons in DMH augment our understanding of the central thermoregulatory circuitry in non-torpid mammals. Our data will contribute to the development of novel therapeutic approaches to induce therapeutic hypothermia for treating drug-resistant fever, and for improving glucose and energy homeostasis.

## Introduction

Core temperature (T_CORE_) in mammals is regulated by the central nervous system (CNS). To maintain T_CORE_ within the normal range, thermal signals from the skin and core are integrated in the preoptic hypothalamus, which contains neurons that control the level of activation of thermoregulatory effectors including brown adipose tissue (BAT) and skeletal muscle shivering for thermogenesis, and cutaneous blood vessels for heat dissipation. Numerous studies have led to a neural circuit model that describes the fundamental CNS pathways underlying the regulation of T_CORE_ in rats^1,2^, as well as those responsible for elevated T_CORE_ during fever in rats^1,3^ and mice^4^.

An increase in the activity of thermogenesis-promoting neurons in the dorsomedial hypothalamus/dorsal hypothalamic area (DMH/DA) is a key feature of the neural circuits responsible for the activation of BAT thermogenesis in response to a cold environment and during the febrile response to infection^3,5^. Both the cold-evoked and febrile increases in the activity of the DMH/DA neurons that drive thermogenesis have been postulated to arise from an excitation-biasing shift in the balance between their excitatory and inhibitory inputs that occurs when the warm-driven, GABAergic inhibitory input from neurons in the medial preoptic area (MPA)^6–9^ to DMH/DA is reduced. Although the cold-evoked and febrile increases in BAT thermogenesis require activation of glutamate receptors in the DMH/DA^10,11^, the sources of the excitatory inputs to the BAT sympathoexcitatory neurons in the DMH/DA remain unknown.

We demonstrate the existence of a novel glutamatergic excitatory input to the DMH/DA from neurons in the median preoptic area (MnPO), which is necessary for the cold-evoked and febrile increases in BAT sympathetic nerve activity (SNA) and BAT thermogenesis. This discovery provides important new insights into the neural circuit mechanisms underlying the thermoregulatory control of BAT thermogenesis and BAT metabolism.

## Results

### Inhibition of neurons in MnPO inhibits febrile BAT thermogenesis elicited by PGE_***2***_ in MPA

**Figure 1:**
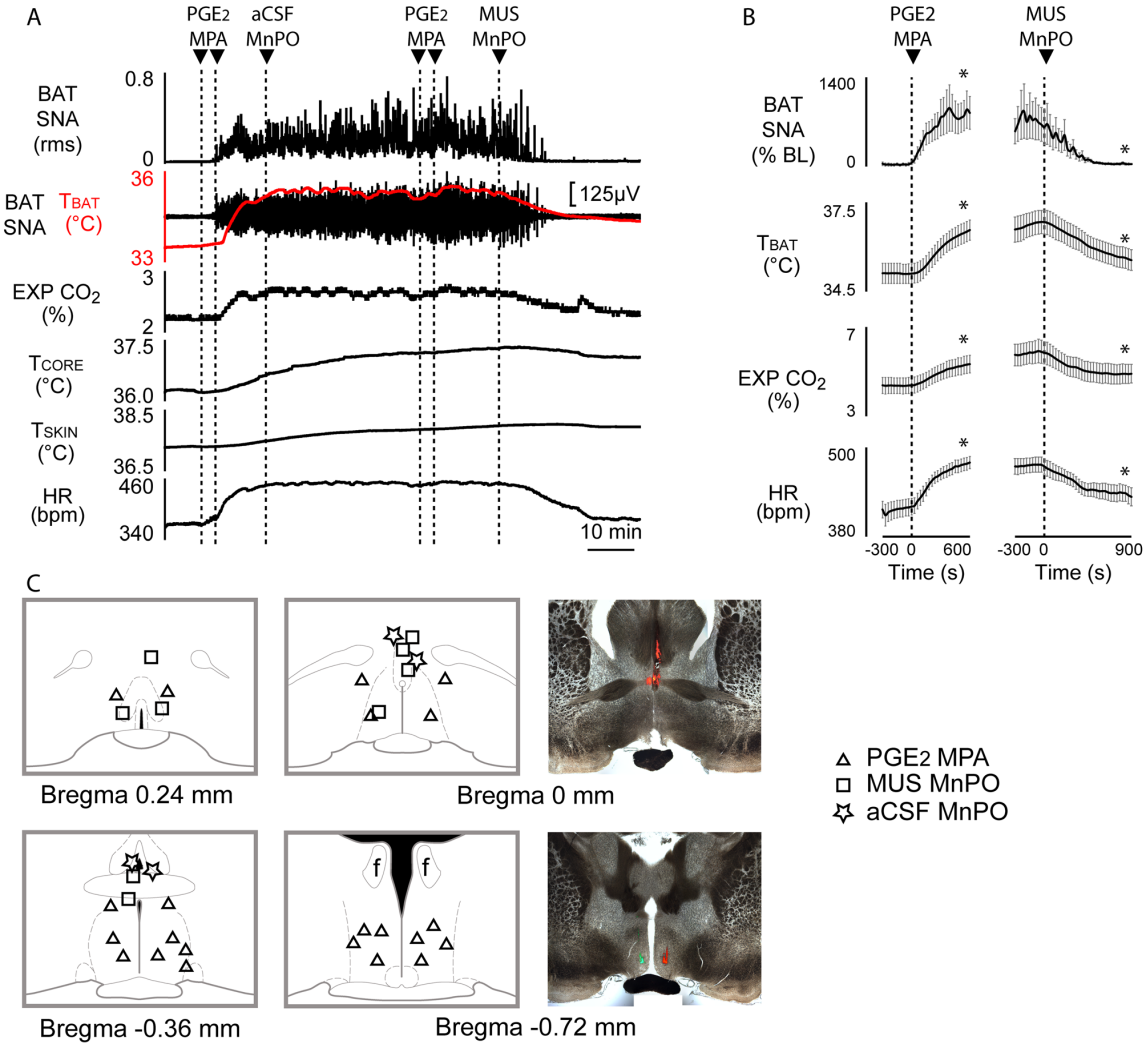
Inhibition of MnPO neurons blocks febrile PGE_2_-induced thermogenesis in MPA. **(A)** Nanoinjection of muscimol (MUS), but not artificial cerebrospinal fluid (aCSF), into MnPO completely reversed the increase in BAT SNA, T_BAT_, and HR evoked by nanoinjection of PGE2 in MPA. **(B)** Left panel shows group data representing the increases in BAT SNA (% BL: % baseline), T_BAT_, expired CO_2_ (EXP CO_2_) and heart rate (HR) following nanoinjection of PGE_2_ into MPA (n = 12). Right panel shows the inhibition of the same variables induced by nanoinjection of MUS in MnPO (n = 9). **(C)** Plots show the location of PGE_2_ in MPA and MUS in MnPO injection sites on schematic drawings through the preoptic hypothalamus. Panels in the right show the histological location of injection sites in MnPO (upper) and MPA (lower) marked with fluorescent beads. *p < 0.05; T_CORE_: core temperature; T_SKIN_: skin temperature.

To determine whether neurons in the MnPO provide a necessary excitation to drive the febrile BAT thermogenic response to central PGE_2_^5^, we inhibited neuronal activity in MnPO after nanoinjecting PGE_2_ into MPA. PGE_2_ into MPA (Fig. 1C) caused a prompt and significant increase in BAT SNA (Fig. 1A,B; pre-PGE_2_: 88.5 ± 55.5%baseline (BL) vs post-PGE_2_ peak: 966.1 ± 317.3% BL, F_(40,11)_ = 7.816, *p* < 0.0001; individual comparison (IC)_(−120 s vs 600 s)_, n = 12, t* = 4.434, *p* < 0.05). This increase in BAT SNA activated BAT thermogenesis resulted in a rise of T_BAT_ (Fig. 1A,B; ∆T_BAT_ =  + 1.45 ± 0.24 °C from a pre-PGE_2_ value of 35.1 ± 0.3 °C, F_(40,11)_ = 38.85, *p* < 0.0001; IC_(−120 s vs 600 s)_, n = 12, t* = 9.095, *p* < 0.05), and an elevated energy expenditure, reflected by an increase in expired CO_2_ (Fig. 1A,B; ∆EXP CO2 =  + 0.9 ± 0.1%, from a pre-PGE_2_ level of 4.4 ± 0.4%, F_(40,11)_ = 45.36, *p* < 0.0001; IC_(−120 s vs 600 s)_, n = 12, t* = 10.17, *p* < 0.05). Activation of BAT thermogenesis was accompanied by tachycardia (Fig. 1A,B; ∆HR =  + 68.5 ± 14.8 bpm from a pre-PGE_2_ level of 410 ± 12.1 bpm, F_(40,11)_ = 19.70, *p* < 0.0001; IC_(−120 s vs 600 s)_, n = 12, t* = 6.922, *p* < 0.05).

Subsequent inhibition of MnPO neurons (Fig. 1A,C) with muscimol reversed both the activation of BAT thermogenesis and the tachycardia elicited by PGE_2_ in MPA (Fig. 1A,B). Muscimol in MnPO decreased the PGE_2_-evoked increase in BAT SNA (Fig. 1A,B; pre-muscimol: 475.2 ± 169.7% BL, nadir: 41.2 ± 7.7% BL, F_(40,8)_ = 4.245, p < 0.0001; IC_(−120 s vs 900 s)_, n = 9, t* = 3.482, *p* < 0.05), T_BAT_ (Fig. 1A,B; ∆T_BAT_ =  − 1.05 ± 0.19 °C, pre-muscimol: 36.6 ± 0.4 °C; F_(40,8)_ = 22.20, *p* < 0.0001; IC_(−120 s vs 900 s)_, n = 9, t* = 9.171, *p* < 0.05) and expired CO_2_ (Fig. 1A,B; ∆EXP CO_2_: − 0.8 ± 0.2%, pre-muscimol: 5.7 ± 0.5%; F_(40,8)_ = 14.05, *p* < 0.0001; IC_(−120 s vs 900 s)_, n = 9, t* = 6.295, *p* < 0.05). Muscimol in the MnPO reduced the PGE_2_-evoked increase in HR (Fig. 1A,B; ∆HR =  − 44 ± 13.4 bpm, pre-muscimol: 472 ± 11.6 bpm; F_(40,8)_ = 6.988, *p* < 0.0001, IC_(−120 s vs 900 s)_, n = 9, t* = 5.143, *p* < 0.05). aCSF injection in MnPO did not affect the PGE_2_-evoked elevation of BAT thermogenesis (Fig. S1).

### Inhibition of MnPO neurons reverses cold-evoked increases in BAT SNA and BAT thermogenesis

**Figure 2:**
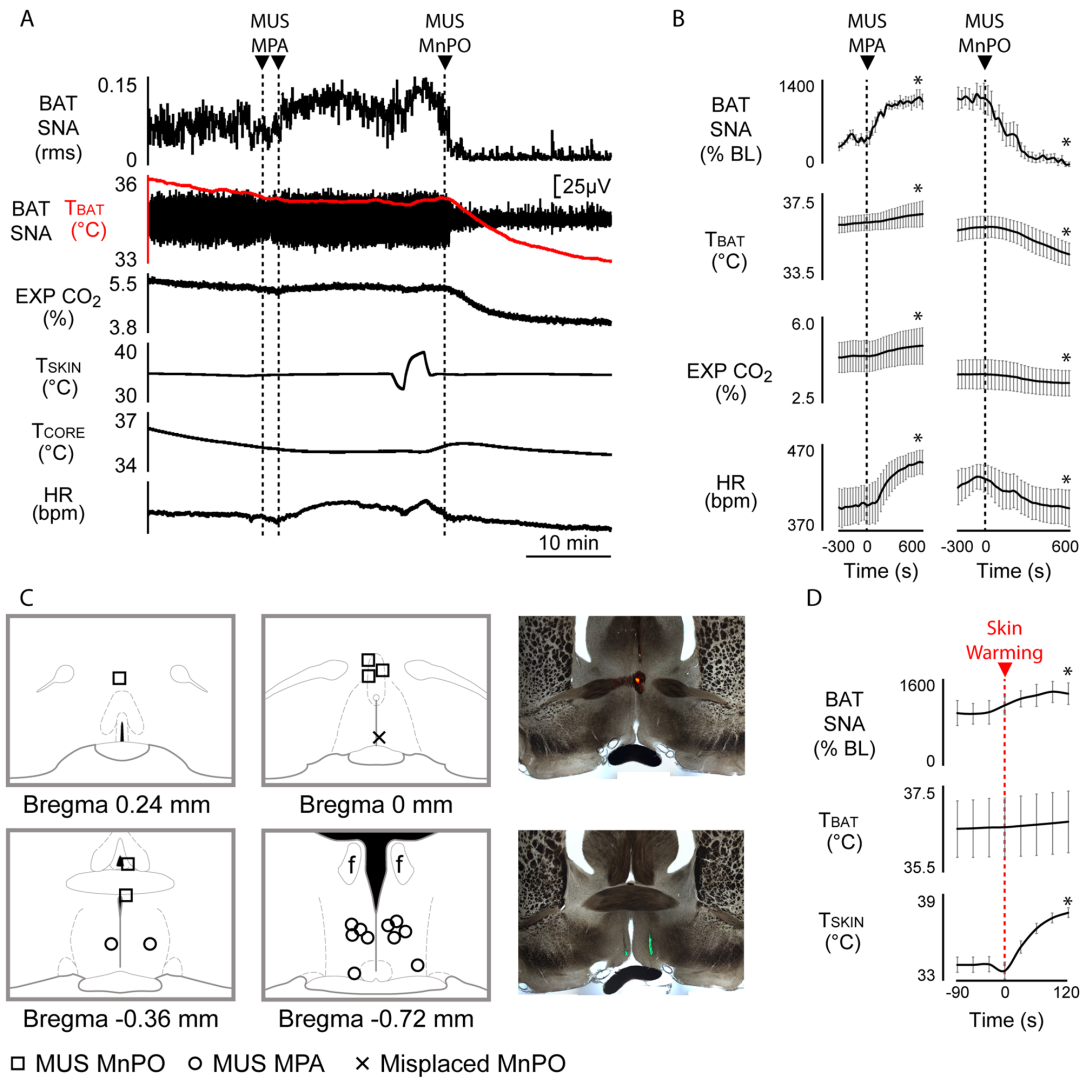
Inhibition of MnPO neurons prevents cold-evoked BAT SNA and thermogenesis. **(A)** Nanoinjection of muscimol (MUS) into MPA elicited an increase in the ongoing cold-evoked BAT SNA and BAT thermogenesis (T_BAT_). During inhibition of neurons in MPA, nanoinjection of MUS into the MnPO completely reversed the cold-evoked BAT SNA, T_BAT_ and expired CO_2_ (EXP CO_2_) and HR. **(B)** Left panel shows group data representing the increases in the ongoing cold-evoked BAT SNA (% BL: % baseline), T_BAT_, EXP CO_2_ and HR in response to nanoinjection of MUS in MPA (n = 6). Right panel shows the effect on the same variables induced by nanoinjection of MUS in MnPO (n = 6). **(C)** Plots depict the location of MUS in MPA and MUS in MnPO injection sites on schematic drawings through the preoptic hypothalamus. Panels in the right show the histological location of injection sites in MnPO (upper) and MPA (lower) marked by fluorescent beads. **(D)** Group data showing that following nanoinjection of MUS in MPA, BAT SNA is no longer inhibited by skin warming (n = 4). *p < 0.05; T_CORE_: core temperature; T_SKIN_: skin temperature.

To determine if the activity of neurons in the MnPO is required for the cold-evoked increases in BAT SNA and BAT thermogenesis, we pretreated the MPA with muscimol to eliminate the activity of GABAergic neurons in MPA, which are postulated to inhibit thermogenesis-promoting neurons in the DMH during skin warming^1,12–14^, and then we inhibited MnPO neurons during skin cooling (Fig. 2A). We reasoned that since skin cooling is postulated to drive a MnPO-mediated inhibition of MPA^15,16^, inhibiting MnPO without a pretreatment inhibition of MPA neurons would not allow us to distinguish between the effect of eliminating MnPO-mediated inhibitory drive to MPA and the effect of eliminating a MnPO-mediated excitatory drive, potentially to DMH neurons, that is necessary for the cooling-evoked activation of BAT SNA. Indeed, inhibition of MnPO without inhibitory pretreatment of MPA does inhibit cold-evoked BAT SNA^16^.

Cooling-evoked BAT SNA and BAT thermogenesis was elicited by reducing T_SKIN_ to 34.7 ± 0.2 °C, which resulted in a mean T_CORE_ of 35.9 ± 0.2 °C. Bilateral nanoinjections of muscimol in the MPA elicited an increase in the cold-evoked level of BAT SNA (Fig. 2A,B; pre-muscimol (i.e., cold-evoked): 278.4 ± 68.0% BL, peak: 1035.0 ± 122.5% BL; F_(40,4)_ = 7.562, *p* = 0.0001; IC_(−120 s vs 600 s)_, n = 5, t* = 5.289, *p* < 0.05), which increased T_BAT_ (∆T_BAT_ =  + 0.5 ± 0.3 °C, pre-muscimol: 36.0 ± 0.4 °C; F_(40,4)_ = 1.897, *p* < 0.0001; IC_(−120 s vs 600 s)_, n = 5, t* = 2.437), and expired CO_2_ (Fig. 2A,B; ∆EXP CO_2_ = 0.4 ± 0.2%, pre-muscimol: 4.4 ± 0.6%; F_(40,4)_ = 2.377, *p* < 0.0001; IC_(−120 s vs 600 s)_, n = 5, t* = 2.302, *p* < 0.05). HR also increased (Fig. 2A,B; ∆HR = 55.6 ± 24.0 bpm, pre-muscimol: 399.6 ± 20.5 bpm; F_(40,4)_ = 7.441, *p* < 0.0001; IC_(−120 s vs 600 s)_, n = 5, t* = 4.665, *p* < 0.05).

Following muscimol nanoinjection in MPA, a bout of skin warming (∆T_SKIN_: + 3.6 ± 0.5 °C, pre-muscimol: 34.1 ± 0.5 °C) increased BAT SNA (Fig. 2A,D; pre-warming: 1026.0 ± 234.6% BL vs. post-warming: 1326.0 ± 199.5% BL; F_(8,3)_ = 6.678, *p* = 0.0001; IC_(−90 s vs 120 s)_, n = 4, t* = 3.963, *p* < 0.05). However, the increase in BAT SNA did not affect T_BAT_ (pre-warming: 36.5 ± 0.7 °C vs post-warming: 36.7 ± 0.8 °C; F_(8,3)_ = 0.7743, *p* > 0.05; IC_(−90 s vs 120 s)_, n = 4, t* = 1.662, *p* > 0.05).

**Figure 3:**
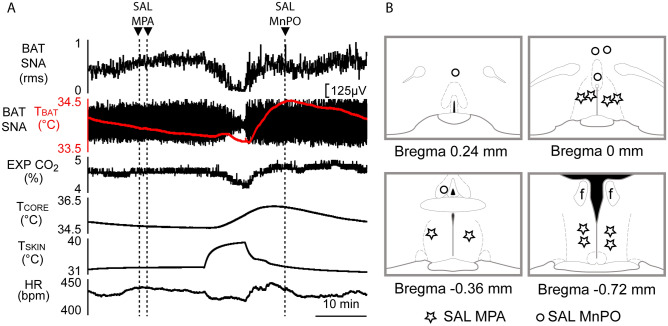
Saline (SAL) vehicle nanoinjections in MPA or in MnPO do not affect BAT SNA or BAT thermogenesis. **(A)** Nanoinjection of SAL in MPA does not affect BAT SNA or prevent the skin-warming induced inhibition of cold-evoked BAT SNA and thermogenesis (T_BAT_: BAT temperature; T_CORE_: core temperature; EXP CO_2_: expired CO_2_; HR: heart rate). **(B)** Location of SAL nanoinjections sites plotted on schematic drawings of the preoptic area at the level of MPA (n = 5, bilateral) and MnPO (n = 5).

Ten minutes after nanoinjection of muscimol in MPA, muscimol nanoinjection in MnPO eliminated the elevated BAT SNA and BAT thermogenesis resulting from both skin/core cooling and inhibition of MPA neurons. Muscimol in MnPO decreased BAT SNA (Fig. 2A,B; pre-muscimol in MnPO: 1082 ± 187.8% BL, nadir: 141.2 ± 194.2% BL; F_(40,4)_ = 25.10, *p* < 0.0001; IC_(−120 s vs 600 s)_, n = 5, t* = 9.063, *p* < 0.05), T_BAT_ (∆T_BAT_ =  − 1.6 ± 0.2 °C, pre-muscimol in MnPO: 35.8 ± 0.5 °C; F_(40,4)_ = 34.7, *p* < 0.0001; IC_(−120 s vs 420 s)_, n = 5, t* = 10.46, *p* < 0.05), expired CO_2_ (Fig. 2A,B; ∆EXP CO_2_ =  − 0.36 ± 0.08%, pre-muscimol in MnPO: 3.7 ± 0.6%; F_(40, 4)_ = 17.92, *p* < 0.0001; IC_(−120 s vs 600 s)_, n = 5, t* = 6.692, *p* < 0.05), and HR (Fig. 2A,B; ∆HR =  − 36.2 ± 12.7 bpm, pre-muscimol in MnPO: 432 ± 14.3 bpm; F_(40,4)_ = 6.570, *p* < 0.0001; IC_(−120 s vs 600 s)_, n = 5, t* = 4.827, *p* < 0.05). Thus, the activity of a population of neurons in the MnPO is required for the skin/core cooling-evoked excitation of BAT SNA, BAT thermogenesis and HR, as well as the additional BAT excitation and tachycardia that follows inhibition of MPA neurons in cool rats. Nanoinjection of saline (vehicle) did not affect any of the thermogenic or cardiovascular variables (Figs. 3 and S2).

### Disinhibition of MnPO neurons after muscimol injection in MPA increases BAT thermogenesis

Having established that the activity of MnPO neurons is required for the excitation of BAT SNA evoked by either skin/core cooling or PGE_2_ in the MPA, we sought to determine if the warm-evoked inhibition of BAT thermogenesis involves both an increase in the MPA GABAergic inhibitory input to DMH^1,5,13^ and a reduction in the excitatory drive for BAT thermogenesis from MnPO. We reasoned that if both are contributing, then the inhibited BAT SNA in warm-exposed rats would be unaffected by injecting muscimol in MPA, but that subsequent disinhibition of MnPO would increase in BAT SNA.

**Figure 4:**
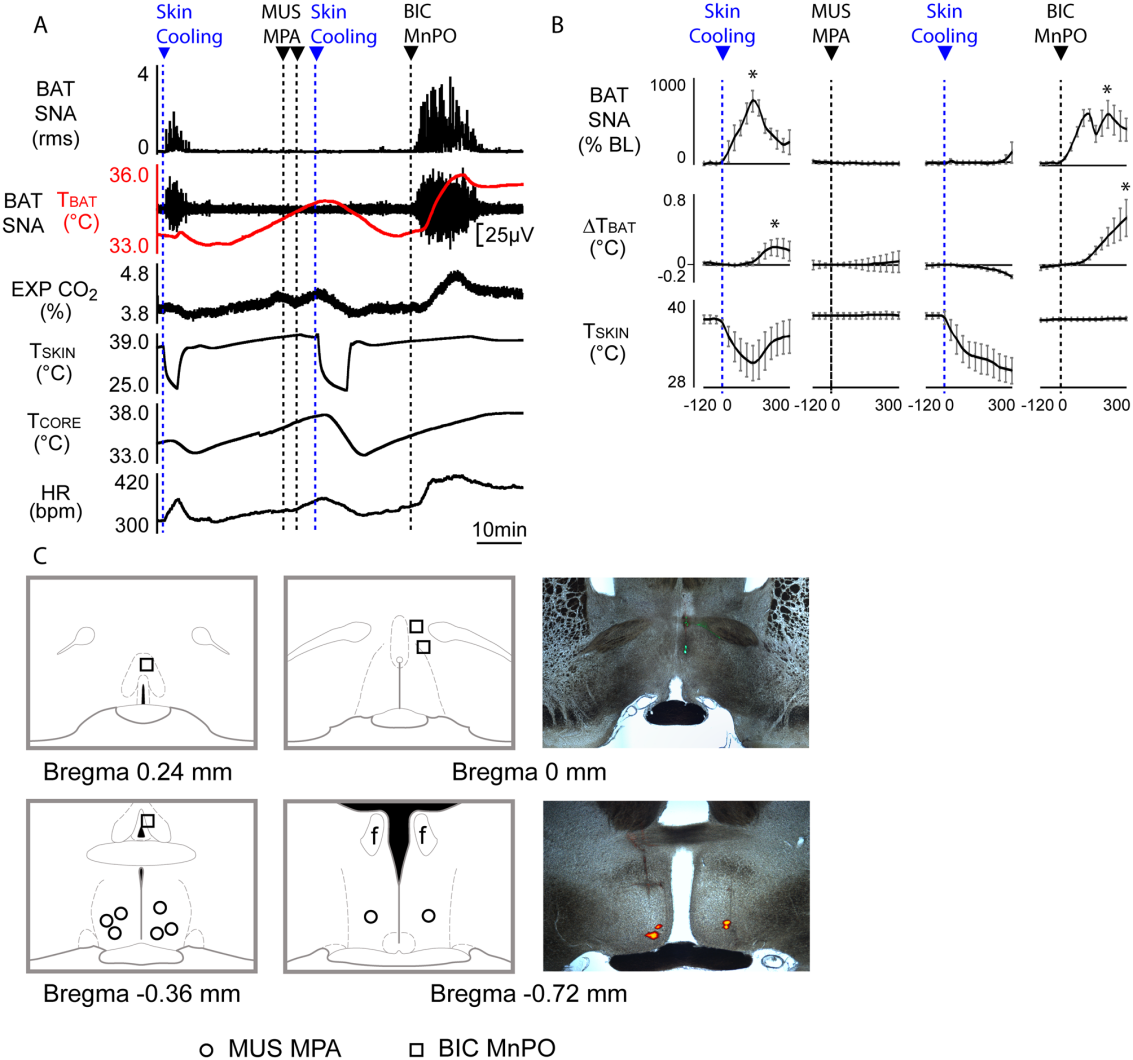
BAT SNA and BAT thermogenesis are increased by nanoinjection of bicuculline (BIC) in MnPO after nanoinjection of muscimol (MUS) in MPA. **(A)** Under warm skin condition and inhibition of BAT SNA, nanoinjection of MUS in MPA did not affect the level of BAT SNA, but prevented cold-evoked increases in BAT SNA, T_BAT_, and HR. Subsequent nanoinjection of BIC in MnPO elicited a prompt increase in BAT SNA and BAT thermogenesis. **(B)** Group data from rats with warm skin demonstrating: (**a**) normal skin cooling-evoked increase in BAT SNA and T_BAT_ (first column, n = 4); (**b**) absence of change in BAT SNA or T_BAT_ following bilateral nanoinjection of MUS into MPA (second column, n = 4); (**c**) inhibition of cold-evoked response following bilateral injection into MPA (third column, n = 4); and (**d**) nanoinjection of BIC in MnPO increases BAT SNA and T_BAT_ (n = 4). **(C)** Plots represent the location of MUS in MPA and BIC in MnPO injection sites on schematic drawings through the preoptic hypothalamus. Panels on the right illustrate the histological location of injection sites in MnPO (upper) and MPA (lower) marked by fluorescent beads. *p < 0.05; T_CORE_: core temperature; T_SKIN_: skin temperature. EXP CO_2_: expired CO_2_.

Warm-evoked inhibition of BAT SNA and BAT thermogenesis was established by maintaining T_CORE_ and T_SKIN_ above 36 °C (Fig. 4A). Muscimol in MPA did not affect the warm-evoked level of BAT SNA (Fig. 4A,B; pre-muscimol in MPA: 14.9 ± 17% BL vs. post-muscimol in MPA: 11.8 ± 8.5% BL, F_(14,3)_ = 1.301, *p* = 0.2473; IC_(−120 s vs 300 s)_, n = 4, t* = 1.836, *p* > 0.05) or T_BAT_ (pre-muscimol in MPA: 36.3 ± 0.8 °C vs. post-muscimol in MPA: 36.3 ± 0.7 °C; F_(14,3)_ = 0.03969, *p* = 1; IC_(−120 s vs 300 s)_, n = 4, t* = 0.4259, *p* > 0.05). However, muscimol in MPA prevented the skin/core cooling-evoked increases in BAT SNA (ΔBAT SNA pre-muscimol in MPA: − 14.7 ± 8.7% BL vs ΔBAT SNA post-muscimol in MPA: − 5.1 ± 13.9% BL; F_(14,3)_ = 0.9144, *p* = 0.5510; IC_(−120 s vs 300 s)_, n = 4, t* = 0.8825, *p* > 0.05) and in BAT thermogenesis (∆T_BAT_ pre-muscimol in MPA: − 0.012 ± 0.035 °C vs ∆T_BAT_ post-muscimol in MPA: − 0.14 ± 0.016 °C; F_(14,3)_ = 5.850, *p* < 0.0001; IC_(−120 s vs 300 s)_, n = 4, t* = 5.268, p < 0.05). The pre- and post-muscimol in MPA skin cooling stimuli were not different (ΔT_SKIN_ pre-muscimol in MPA: − 6.0 ± 2.2 °C vs ΔT_SKIN_: post muscimol in MPA: − 7.6 ± 1.9 °C; n = 4, t-test t = 1.089, *p* = 0.1779). This result indicates that, in contrast to cool rats (Fig. 2), inhibition of MPA neurons in warm rats is not sufficient to allow cooling-evoked increases in BAT SNA and BAT thermogenesis.

Following muscimol in MPA, nanoinjection of bicuculline in MnPO to block local GABA_A_ receptors elicited an increase in BAT SNA (pre-bicuculline in MnPO: 20.5 ± 22.7% BL vs. post-bicuculline in MnPO: 508.7 ± 160.1% BL, F_(14,3)_ = 7.206, *p* < 0.0001; IC_(−120 s vs 300 s)_, n = 4, t* = 3.329, *p* < 0.05) and in T_BAT_ (pre-bicuculline in MnPO: 35.8 ± 0.8 °C vs. post-bicuculline in MnPO: 36.4 ± 0.7 °C, F_(14,3)_ = 6.407, *p* < 0.0001; IC_(−120 s vs 300 s)_, n = 4, t* = 5.196, *p* < 0.05). This result indicates that, in warm rats, there is a GABAergic input to neurons in the MnPO that is not only active after inhibition of neuronal activity in the MPA but is also necessary and sufficient to sustain the warm-evoked inhibition of BAT SNA after inhibition of MPA neurons. Further, the increase in BAT SNA after blocking a GABAergic input to MnPO is consistent with the existence of a population of BAT sympathoexcitatory neurons in the MnPO.

### BAT thermogenesis elicited by activation of MnPO neurons or by skin cooling requires a glutamatergic input to BAT sympathoexcitatory neurons in DMH

**Figure 5:**
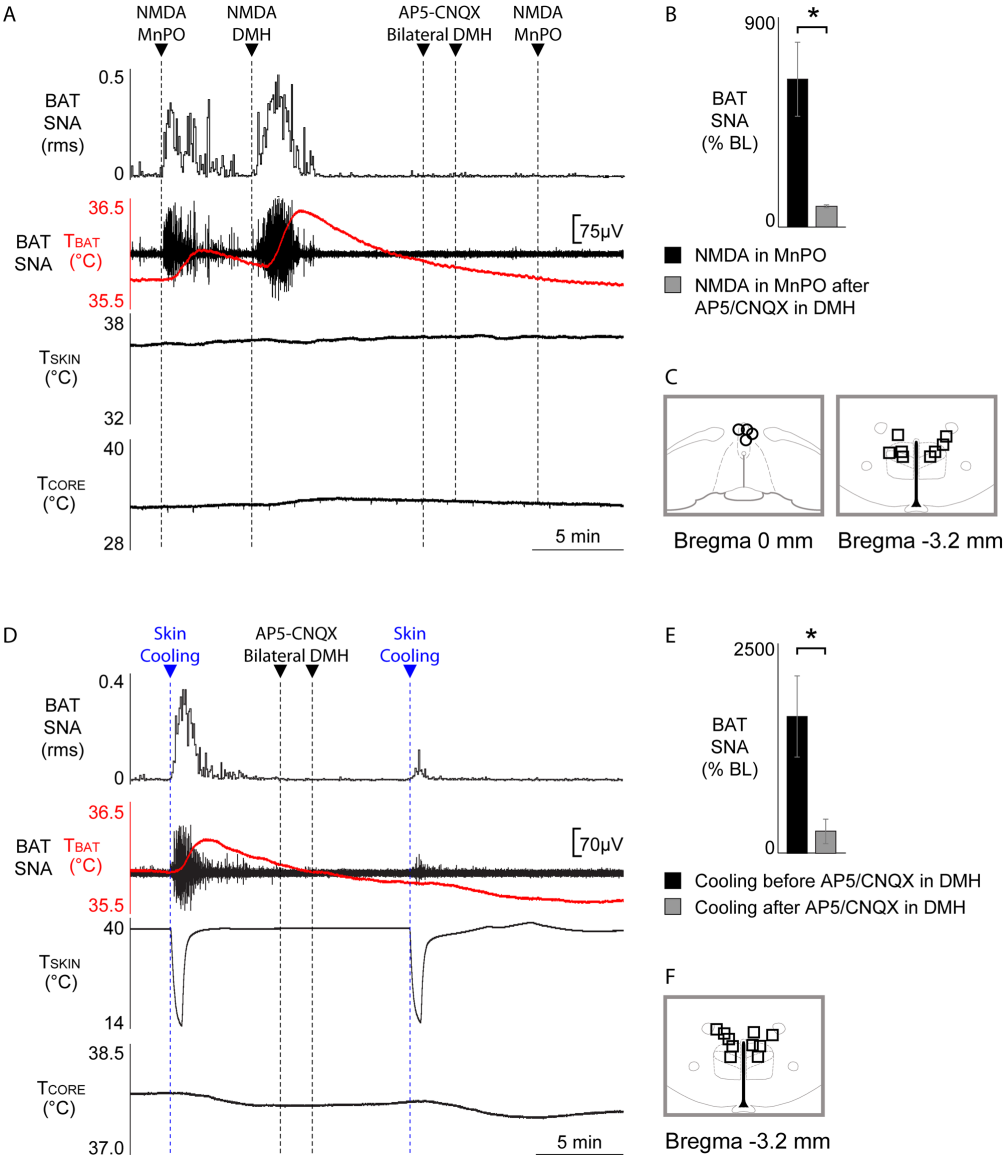
Antagonism of glutamate receptors in DMH prevents the stimulation of BAT SNA and BAT thermogenesis elicited by nanoinjection of NMDA in the MnPO (**A**, **B**) or by skin cooling (**D**, **E**). **(A)** Under warm skin condition and inhibition of BAT SNA, nanoinjection of NMDA in MnPO elicited a prompt increase in BAT SNA and T_BAT_. Nanoinjection of AP5/CNQX into the DMH prevented the increases in BAT SNA and T_BAT_ from nanoinjection of NMDA in the MnPO. NMDA injection in DMH (to trigger thermogenic response) was used to confirm that the AP5/CNQX injections were done in the appropriate DMH location, which contains the sympathoexcitatory neurons involved in BAT thermogenesis. **(B)** Group data from rats with warm skin demonstrating: (**a**) increase in BAT SNA in response to nanoinjection of NMDA in MnPO (n = 4), and (**b**) that bilateral nanoinjection of AP5/CNQX into DMH blocked the increase in BAT SNA from nanoinjection of NMDA in MnPO (n = 4). **(C)** Plots show NMDA in MnPO and AP5/CNQX in DMH injection sites on schematic drawings through the hypothalamus. **(D)** Under warm skin condition and inhibition of BAT SNA, skin cooling elicited a prompt increase in BAT SNA and T_BAT_. Nanoinjection of AP5/CNQX into the DMH prevented cold-evoked increases in BAT SNA and T_BAT_. **(E)** Group data from rats with warm skin demonstrating: (**a**) increase in BAT SNA in response to skin cooling (n = 5), and (**b**) that bilateral nanoinjection of AP5/CNQX into DMH blocked cold-evoked BAT SNA (n = 5). **(F)** Plots show AP5/CNQX in DMH injection sites on schematic drawings through the hypothalamus. *p < 0.05; T_CORE_: core temperature; T_SKIN_: skin temperature.

Next, we sought to determine if the BAT sympathoexcitatory output from a population of MnPO neurons depends on a glutamatergic excitation of DMH neurons. In warm rats with low levels of BAT SNA (Fig. 5A), nanoinjection of NMDA into MnPO activated BAT SNA (Fig. 5A,B; pre-NMDA in MnPO: 192.4 ± 43.1% BL vs. post-NMDA in MnPO: 634.1 ± 158.7% BL, n = 4, t = 3.668, *p* = 0.0175). Subsequently, nanoinjections of AP5/CNQX in BAT sympathoexcitatory sites in the DMH (Fig. 5A,B,C) prevented the NMDA in MnPO-evoked activation of BAT SNA (Fig. 5B; ΔBAT SNA pre-AP5/CNQX in DMH: 634.1 ± 158.7% BL vs. ΔBAT SNA post-AP5/CNQX in DMH: 89.08 ± 6.9% BL, n = 4, t = 3.323, *p* = 0.0225). Nanoinjections of AP5/CNQX in the DMH (Fig. 5F) also prevented the cold-evoked activation of BAT SNA (Fig. 5D,E; ΔBAT SNA pre-AP5/CNQX in DMH: 1635.4 ± 484.2% BL vs ΔBAT SNA post-AP5/CNQX in DMH: 258.2 ± 147.7% BL, n = 5, t = 2.932, *p* = 0.0214). These results indicate not only that activating neurons in the MnPO drives increases in BAT SNA and BAT thermogenesis, but also that the BAT sympathoexcitatory responses to either skin cooling or activation of neurons in MnPO similarly require an active glutamatergic input to the DMH.

### Cold-evoked increased BAT thermogenesis is eliminated by brain transections rostral to DMH

**Figure 6:**
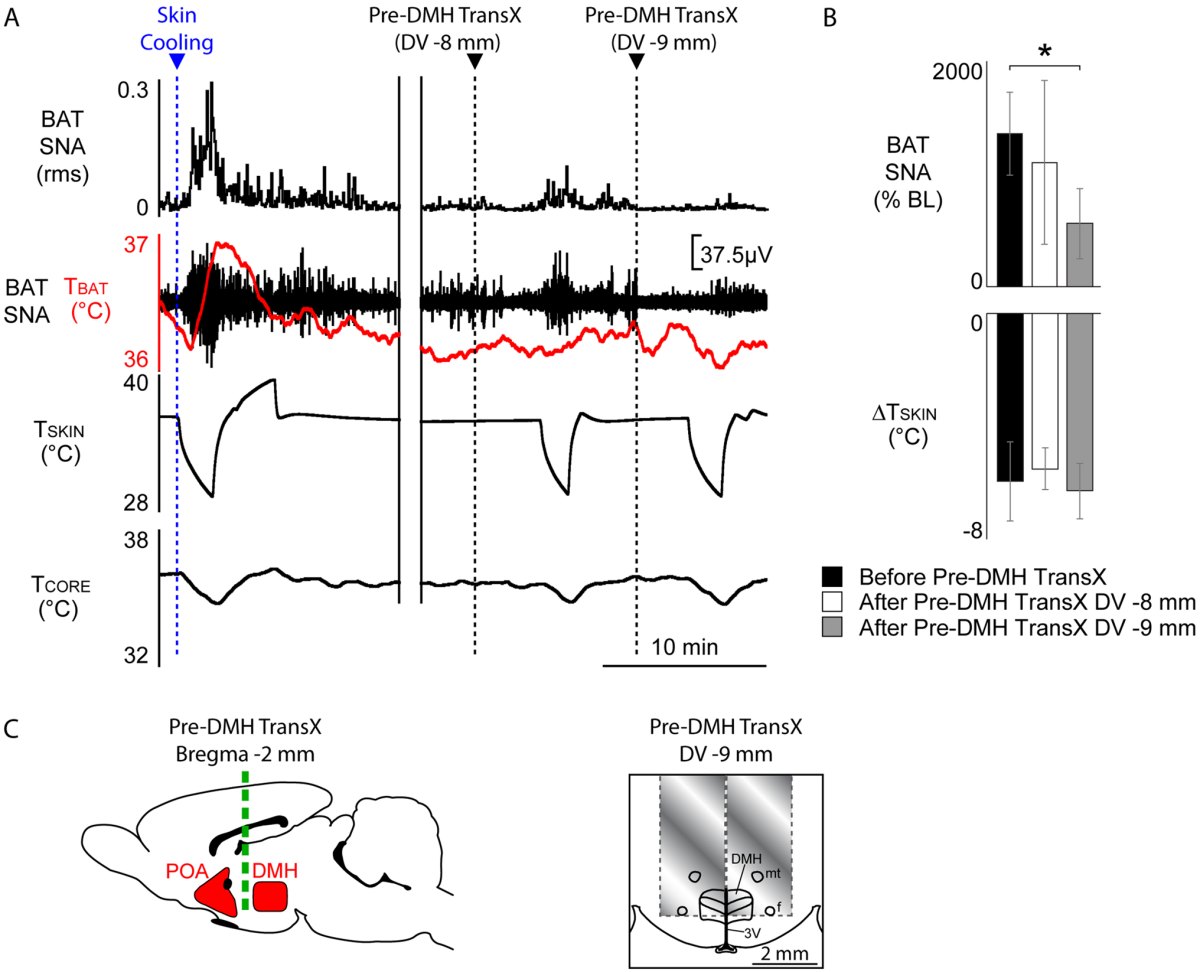
Midline brain transection rostral to DMH eliminates cold-evoked increases in BAT SNA. **(A)** Skin cooling elicited the normal increase in BAT SNA and T_BAT_. A brain transection just rostral to DMH (Pre-DMH TransX, bregma − 2 mm), extending bilaterally 2 mm on both sides of the midline and to a dorsoventral (DV) depth of − 9 mm (but not − 8 mm) below the dorsal brain surface, significantly reduced the skin cooling-evoked increase in BAT SNA. **(B)** Group data demonstrating: (**a**) normal increase in BAT SNA in response to skin cooling (n = 4); (**b**) Pre-DMH TransX to a depth of − 8 mm below the brain surface does not affect the BAT SNA response to skin cooling (n = 4); and (**c**) Pre-DMH TransX to a depth of − 9 mm below the brain surface reduces the BAT SNA response to skin cooling (n = 4). **(C)** Schematic representations of Pre-DMH TransX illustrating their locations relative to the POA and DMH (sagittal view, left panel) and the approximate area transected by inserting the knife bilaterally to a − 9 mm depth (coronal view, right panel). *p < 0.05; T_CORE_: core temperature; T_SKIN_: skin temperature; f: fornix; mt: mammillothalamic tract; 3 V: third ventricle.

To support the existence of a BAT sympathoexcitatory pathway between the MnPO and the DMH, we used a brain transection approach (Fig. 6C) to determine if the neural pathways traversing the region immediately rostral to the DMH are functionally significant for the skin cooling-evoked stimulation of BAT SNA. An initial transection was made to a depth of − 8.0 mm ventral to the dorsal surface of the brain, and subsequently advanced to a depth of − 9.00 mm, with tests of the BAT SNA response to skin cooling after each depth (Fig. 6A). In warm rats with inhibited BAT SNA (Fig. 6A), skin cooling of − 6.0 ± 1.4 °C (from a baseline of 36.4 ± 0.5 °C) produced the normal cold-defensive increase in BAT SNA (Fig. 6A,B; pre-cooling BAT SNA: 116.9 ± 14.5% BL vs. cooling-evoked peak of 1374.5 ± 373.9% BL, n = 4, t = 3.335, *p* < 0.05). Bilateral brain transections to a depth of − 8 mm had no effect on the skin cooling-evoked increase in BAT SNA (Fig. 6A,B; intact cooling ΔBAT SNA: 1257.6 ± 377.10% BL vs. post − 8 mm transection ΔBAT SNA: 955.6 ± 732.2% BL, F = 4.602, *p* = 0.0383; n = 4, t* = 0.6680, *p* > 0.05). Extension of the transection depth to − 9 mm significantly reduced the cold-evoked increase in BAT SNA (Fig. 6A,B; intact cooling ΔBAT SNA: 1257.6 ± 377.10% BL vs. post − 9 mm transection ΔBAT SNA: 444.5 ± 302.2% BL, F = 4.602, p = 0.0383; n = 4, t* = 2.897, *p* < 0.05). There were no differences among the amplitudes of the skin cooling stimuli employed for the 3 sets of skin cooling trials (F_(11,2)_ = 0.9573, *p* = 0.43). This finding indicates that a pathway(s) traversing the region immediately rostral to the DMH and at a depth greater than 8 mm ventral to the dorsal surface of the brain is necessary for the skin-cooling-evoked increase in BAT SNA.

### Distribution of cold- and warm-activated POA neurons that project to DMH or rostral raphe pallidus (rRPa)

**Figure 7:**
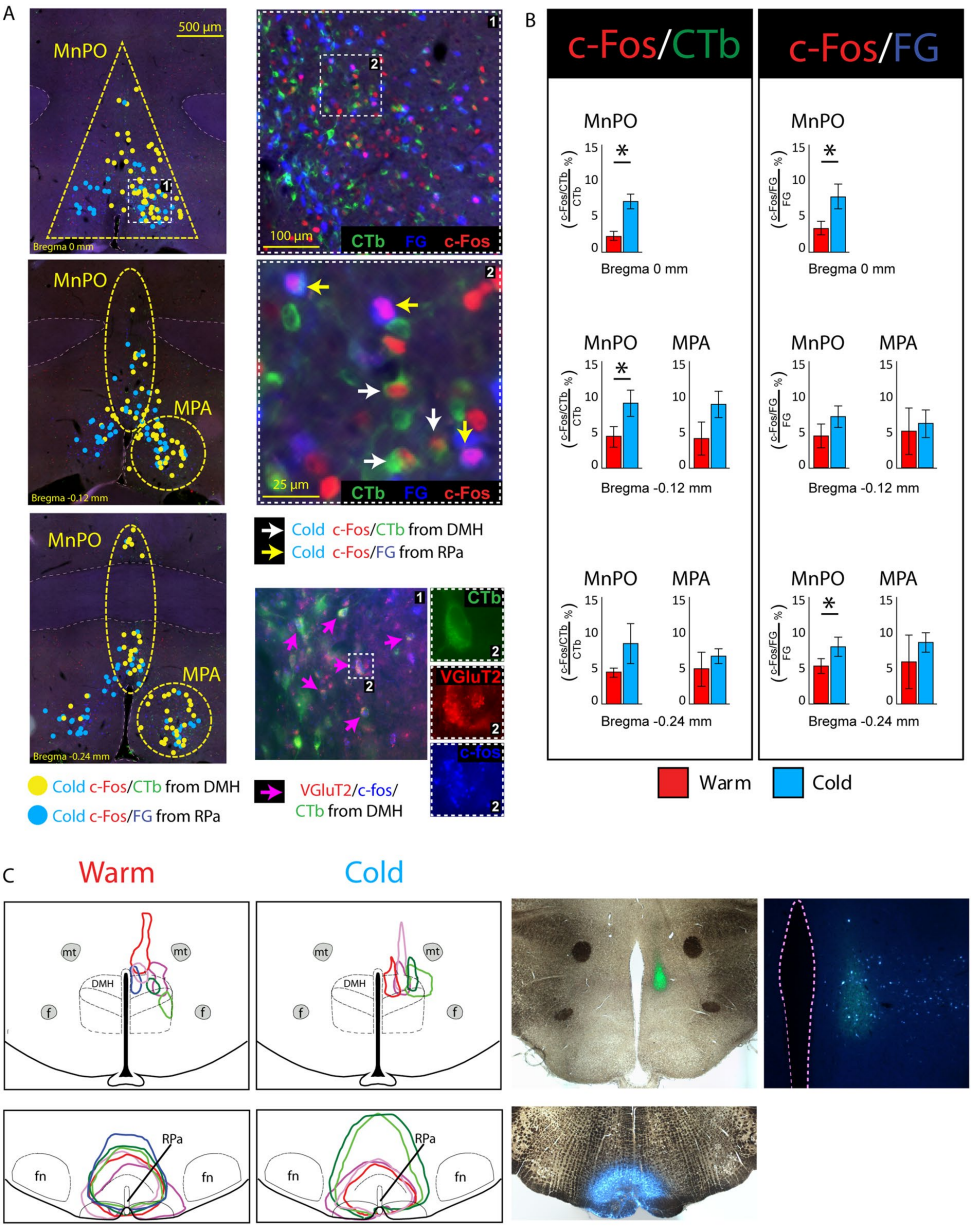
Cold exposure increases c-Fos expression in MnPO neurons that project to DMH and to rRPa. **(A) Left column**: distribution of neurons double-labeled for cold-evoked c-Fos and retrogradely transported CTb from DMH (yellow dots), and for cold-evoked c-Fos and retrogradely transported FG from rRPa (blue dots) at 3 rostrocaudal levels through the MnPO and MPA. Dash yellow lines depict the MnPO and MPA counting areas used for quantitative analysis (panel **B**). **Right column**: top panel shows CTb-ir, FG-ir and c-Fos-ir neurons in the MnPO (high magnification of dashed square 1 in the left column top panel). The middle panel is a high magnification of dashed square 2 in the right column, top panel). White arrows: c-Fos/CTb double-labeled neurons in MnPO; yellow arrows: c-Fos/FG double-labeled neurons. The bottom panel shows neurons in the MnPO triple-labeled for CTb, c-fos and VGluT2 (left image, pink arrows) and a high magnification view of the individual channels of a triple-labeled neuron (from dashed square 2 in the left column, bottom panel, left image). **(B)** Percentage of retrogradely-labeled neurons at the 3 levels of MnPO and MPA shown in (**A**) that also exhibited c-Fos during cold (blue) or warm (red) exposure. Left panel: at the rostrocaudal levels of bregma 0.0 mm and − 0.12 mm, a greater percentage of CTb-ir neurons retrogradely-labeled from the DMH expressed c-Fos during cold exposure than during warm exposure. Right panel: at the rostrocaudal levels of bregma 0.0 mm and − 0.24 mm, a greater percentage of FG-ir neurons retrogradely-labeled from the rRPa expressed c-Fos during cold exposure than during warm exposure. **(C)** In the left panels, the location and extent of the injection sites of CTb in DMH and FG in rRPa, in warm- or cold-exposed rats, are depicted on drawings through the hypothalamus and the medulla. The panels on the right show histological sections illustrating the typical injection sites of CTb in DMH and of FG in rRPa. The far right image shows that the CTb injection in DMH overlaps with a cluster of FG-ir neurons that projects to rRPa. f: fornix; mt: mammillothalamic tract; fn: facial nucleus.

Since activation of BAT sympathoexcitatory neurons in the DMH and rRPa is required for the cold-evoked increases in BAT thermogenesis in rats^1^ and since both of these regions receive inputs from neurons in MnPO and MPA^12,17,18^, we sought to determine if cold exposure activates the MnPO neurons projecting specifically to DMH and/or to rRPa. Two groups of rats were injected with FluoroGold (FG) in the rRPa and cholera toxin subunit B (CTb) in the right DMH, and subsequently exposed to either a warm (T_AMB_: 30 °C, n = 6) or a cold (T_AMB_: 10 °C, n = 5) environment to elicit c-Fos expression indicating neuronal activation. FG nanoinjections were centered on and encompassed, but were not restricted to, the rRPa area of the ventromedial medulla (Fig. 7C, lower left) containing BAT sympathetic premotor neurons^19^ whose activation is required for cold-evoked increases in BAT SNA^20^. Importantly, our CTb injections in the DMH (Fig. 7C, upper left) overlapped with the restricted DMH region containing neurons retrogradely-labeled from the FG injections in the rRPa (Fig. 7C, upper right).

The distributions of CTb-immunoreactive (-ir), FG-ir neurons and c-Fos-ir neurons in both the MnPO and MPA regions (Fig. 7A) were consistent with previous results^12^ (see S3).

In both MnPO and MPA, many of the CTb-ir neurons projecting to DMH were also c-Fos-ir after exposure to either the cold or the warm T_AMB_. In the MnPO (Fig. 7A), a greater percentage of CTb-ir neurons expressed c-Fos after cold exposure than after warm exposure at the level of bregma (Fig. 7B; cold: 7.1 ± 1.0% vs. warm: 2.3 ± 0.7%, n = 11, t = 4.107 *p* = 0.0023) and at − 0.12 mm caudal to bregma (Fig. 7B; cold: 9.2 ± 1.8% vs. warm: 4.4 ± 1.4%, n = 11, t = 4.107 *p* = 0.0023).

In both MnPO and MPA, there were numerous FG-ir neurons (projecting to rRPa) that were also c-Fos-ir after either cold or warm exposure. Only in the MnPO (Fig. 7A) at the level of bregma (Fig. 7B; cold: 7.7 ± 1.7% vs. warm: 3.4 ± 0.9%, n = 11, t = 3.004, *p* = 0.0099) and at − 0.24 mm caudal to bregma (Fig. 7B; cold: 8.1 ± 1.4% vs. warm: 5.4 ± 1.0%, n = 11, t = 1.955 *p* = 0.0457) the percentage of rRPa-projecting neurons that were activated by cold exposure was greater than that activated by warm exposure. In both MnPO and MPA, only a few of the double-labeled (CTb-ir and FG-ir) neurons were also c-Fos-ir after either cold or warm exposure; the numbers of triple-labeled neurons were not different between cold- and warm-exposed rats in any analyzed region (see S4). Together, these data indicate that both the MnPO and the MPA contain cold-activated neurons that project either to the DMH or to the rRPa, but that MnPO and MPA neurons with axons projecting to both thermogenesis-promoting targets are unlikely to make a significant contribution to the physiological responses evoked by cold exposure.

### Cold exposure activates glutamatergic neurons in MnPO that project to DMH

Our physiological results strongly support the requirement for a glutamatergic input from MnPO to the BAT sympathoexcitatory neurons in DMH in the cold-evoked and febrile activations of BAT SNA. To provide anatomical support for such a pathway, we combined in situ hybridization (RNAScope) for VGluT2 mRNA to label glutamatergic neurons in MnPO and c-fos mRNA to label neurons activated by cold exposure, with CTb immunofluorescence to label neurons that project to DMH. We observed several CTb-ir and VGluT2/c-fos-positive neurons in MnPO, intermixed with CTb-ir and VGluT2-negative neurons and non-CTb-ir and VGluT2-positive neurons (Fig. 7A). The combination of immunofluorescence for CTb with RNAScope, which involves protease steps, substantially decreases the immunofluorescence labeling, thereby precluding a reliable quantification of these triple-labeled neurons. Nevertheless, these data demonstrate that the rat MnPO contains a population of glutamatergic, DMH-projecting neurons that is activated by cold exposure.

## Discussion

**Figure 8:**
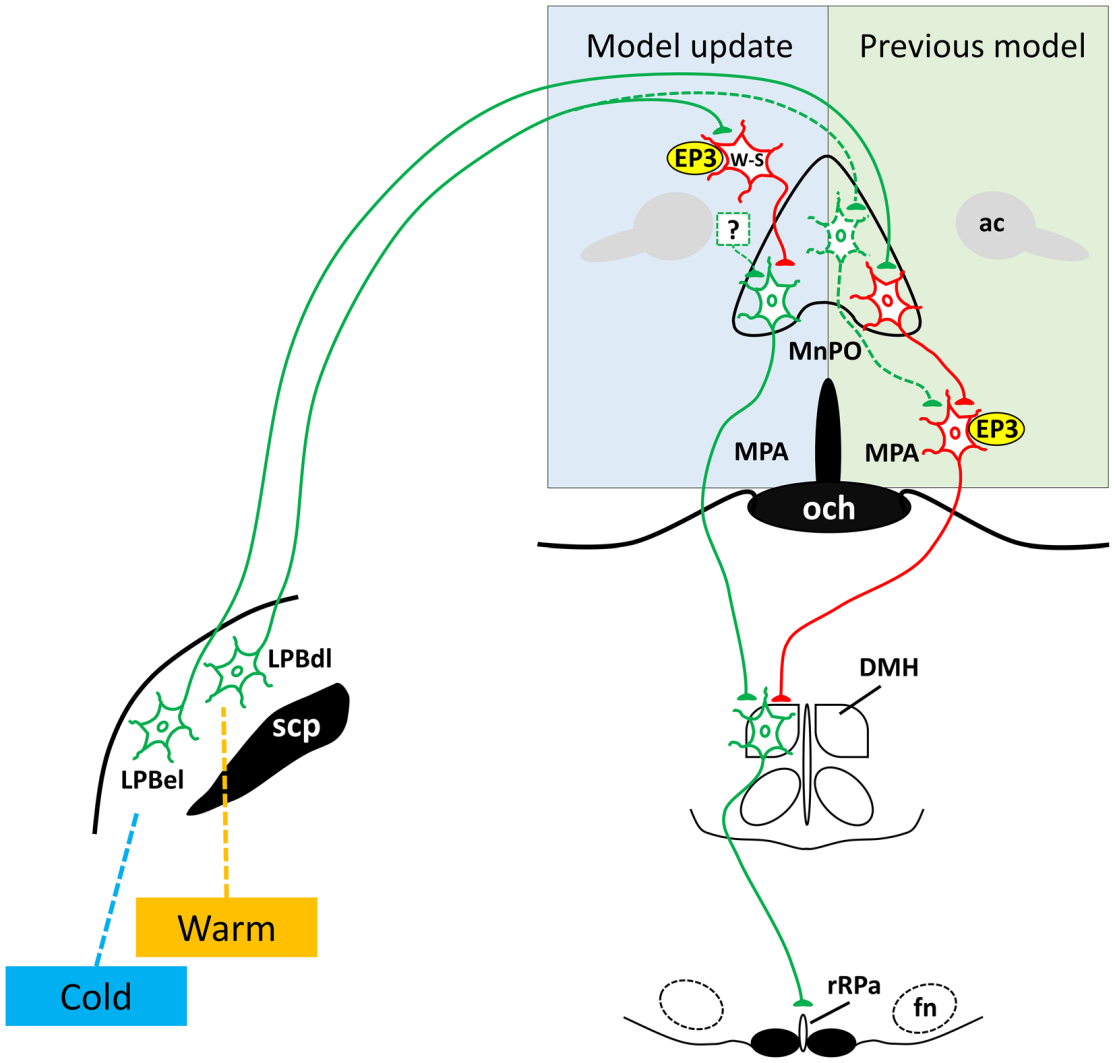
Update of the previous model for the central thermoregulatory network in the rat POA. This thermoregulatory circuit model in rat shows the excitatory (green) and inhibitory (red) neuronal pathways required for the modulation of BAT thermogenesis premotor neurons in rRPa. **In warm defense**, we postulate two factors that reduce the activity of BAT sympathoexcitatory neurons in the DMH and thereby diminish BAT thermogenesis. First, as depicted in the model update, the essential MnPO to DMH glutamatergic drive receives a core-warming activated, GABAergic inhibition from local, warm-sensitive (W-S) neurons whose activity may be augmented by cutaneous warm thermal excitation via the LPBdl. Skin warming-activated LPBdl neurons may also reduce DMH BAT sympathoexcitatory neuronal activity by driving a glutamatergic excitation of MPA GABAergic neurons that project to DMH^1,19,34^. Secondly, skin warming would reduce the excitatory input from skin cooling-activated neurons in the external lateral parabrachial nucleus (LPBel) to GABAergic MnPO neurons that inhibit GABAergic neurons in the MPA, thereby increasing their inhibition of DMH BAT sympathoexcitatory neurons and reducing BAT thermogenesis. In the previous model^16,42^, **during cold defense**, skin cooling leads to activation of glutamatergic neurons in the LPBel that excite MnPO GABAergic neurons which inhibit MPA GABAergic neurons projecting to DMH, resulting in disinhibition of BAT sympathoexcitatory neurons in the DMH, and increasing their activity due to their excitation from the novel MnPO glutamatergic input to DMH shown in the model update. During cold exposure, the reduced warm thermoreceptor discharge and the decreases in T_CORE_ result in disinhibition of both the BAT sympathoexcitatory neurons in the DMH and their glutamatergic excitatory input from the MnPO, thereby increasing the excitatory drive to BAT premotor neurons in rRPa. **The PGE**_**2**_**-mediated febrile increase in BAT thermogenesis** arises from a combination of (**a**) the EP3 receptor-mediated inhibition of GABAergic, W-S neurons in the MnPO, thereby disinhibiting the glutamatergic neurons in the MnPO that provide an excitatory drive to DMH, and (**b**) the EP3 receptor-mediated inhibition of the GABAergic neurons in the MPA that directly inhibit the DMH BAT sympathoexcitatory neurons. fn: facial nucleus; och: optic chiasm; ac: anterior commissure; scp: superior cerebellar peduncle.

To understand the mechanisms underlying the cold-evoked and febrile activations of BAT thermogenesis and BAT energy expenditure, it is necessary to identify the elements of the CNS circuitry mediating these perturbations to metabolic homeostasis. One of the most prominent lacunae in the current model of the CNS regulation of the sympathetic outflow to BAT^21^ is the source(s) of the excitatory input(s) to the thermogenesis-promoting neurons in the DMH that are required for BAT thermogenesis^6,9,11,20,22–25^. The present study provides evidence that glutamatergic neurons in the MnPO provide an excitatory drive to the BAT sympathoexcitatory neurons in the DMH that is required for the activation of BAT SNA and BAT thermogenesis, that occurs during cold defense in rats. We demonstrate that a direct glutamatergic pathway from the MnPO to the DMH is activated during cold exposure, and that antagonism of glutamate receptors in DMH or transection of the pathways between the MnPO and the DMH prevents cold-evoked increases in BAT SNA. Activation of neurons in the MnPO, which would include those glutamatergic DMH projection neurons excited during cold exposure, robustly stimulates BAT SNA and BAT thermogenesis, and both the NMDA in MnPO-evoked and the cold-evoked increases in BAT activity are eliminated by blockade of ionotropic glutamate receptors in the DMH. Inhibition of neurons in the MnPO reverses the increases in BAT SNA evoked by skin cooling, as well as those evoked by nanoinjection of PGE_2_ into the MPA. Together, these data support the novel localization within the MnPO of a critical glutamatergic excitatory input to BAT sympathoexcitatory neurons in the DMH (Fig. 8), thereby expanding our understanding of the fundamental brain pathways that drive BAT thermogenesis for cold defense in the rat^21^.

To activate cold defensive thermogenesis, skin cooling-responsive neurons in the lateral parabrachial nucleus (LPB) provide a glutamatergic excitation to neurons in the MnPO^26,27^, and an excitatory input(s) activates thermogenesis-promoting neurons in the DMH which excite BAT sympathetic premotor neurons in rRPa to drive the sympathetic activation of BAT^6,20,22^. Our study identifies the MnPO as a source of the glutamatergic excitation of BAT sympathoexcitatory neurons in the DMH (Fig. 8) that is necessary for cold defensive thermogenesis.

Our novel finding that inhibition of neuronal activity in the MnPO prevents cold-evoked BAT thermogenesis indicates that a population of neurons within MnPO must be active in order to drive this BAT thermogenic response. Since cold-evoked BAT activation also requires activation of neurons in the DMH and in the rRPa^5,20,28^, and cold exposure activates MnPO neurons that project to these two sites of BAT sympathoexcitatory neurons, and a partial transection just rostral to the DMH eliminates cold-evoked BAT activity, the simplest model to explain these results is that MnPO contains BAT sympathoexcitatory neurons that project to and excite neurons in either DMH or rRPa. However, we extended the earlier observation that NMDA-evoked activation of neurons in the MnPO increases BAT SNA^16^ to show that this MnPO-evoked, as well as the cold-evoked activation of BAT is dependent upon ionotropic glutamate receptors in the DMH. Thus, our data support a model in which the DMH is the target of a glutamatergic projection from the MnPO that drives the BAT thermogenic response to cold exposure (Fig. 8). Indeed, we also demonstrated the existence of just such a cold-activated, glutamatergic pathway from the MnPO to the DMH. Caveats to our conclusion include the possibility that the cold-activated, glutamatergic neurons in MnPO that project to the DMH are mediating other non-BAT physiological responses to cold exposure, and that the cold-evoked and MnPO-evoked activations of BAT thermogenesis could be mediated indirectly by MnPO neurons that project to and excite another, as yet undescribed, glutamatergic input to the BAT thermogenesis-promoting neurons in the DMH.

The level of activity of the BAT sympathoexcitatory neurons in the DMH is determined by the balance of their active excitatory and inhibitory inputs, such that removal of an active inhibitory input (i.e., disinhibition) to the DMH would be a condition supporting increases in BAT SNA and BAT thermogenesis. The earlier finding that the stimulation of BAT activity evoked by activating neurons in the MnPO is reversed by blocking GABA_A_ receptors in MPA^16^, a subregion of the POA where neuronal activation inhibits BAT SNA^10^, led to the proposal that the cooling-evoked activation of BAT involves a disinhibition of the BAT sympathoexcitatory neurons in the DMH^16^. The mouse MPA also contains GABAergic neurons which are activated by skin warming and whose exogenous activation reduces body temperature by inhibiting thermogenesis-promoting neurons in the DMH^6,25^. Also consistent with this disinhibitory model, we found that inhibition of local neurons in the MPA during skin/core cooling led to an additional increase in BAT SNA. Thus, considering this postulated circuitry, simply inhibiting the activity of GABAergic neurons in MnPO would be expected to disinhibit GABAergic neurons in the MPA thereby leading to increased inhibition of BAT sympathoexcitatory neurons in the DMH and a blockade of the cold-evoked activation of BAT^16^. We eliminated the potential contribution that disinhibiting GABAergic MPA neurons might have made to the cooling-evoked activation of DMH neurons by putting a strong inhibition of MPA neurons in place prior to nanoinjecting muscimol in MnPO. This allows us to conclude that the cooling-evoked increase in the activity of BAT sympathoexcitatory neurons in the DMH arises from the combined effect of an active glutamatergic excitation from neurons in the MnPO and a reduced GABAergic inhibition from neurons in the MPA. Since a nearly identical pathway underlies cold-evoked shivering in skeletal muscle^5^, the primary source of thermogenesis in humans^29^, we postulate that the MnPO also contains neurons that provide an essential excitatory input to shivering-promoting neurons in the DMH.

In both the rat and mouse, fever arises from the action of PGE_2_ on prostaglandin E type 3 (EP3) receptors located on neurons throughout the MPA and MnPO^4,30,31^. Similar to cold defense, we found that inhibition of neuronal activity in the MnPO reversed the febrile activation of BAT thermogenesis elicited by PGE_2_ in the MPA, indicating that the activity of a population of neurons in the MnPO is required for the BAT activation in fever. It is unlikely that these MnPO neurons, whose activity is required for febrile thermogenesis in the rat, express the EP3 receptor; since PGE_2_ inhibits EP3 receptor-expressing neurons, such neurons would already be inhibited by PGE_2_. Also similar to cold defense, the PGE_2_-evoked activation of BAT thermogenesis in the rat requires a glutamate receptor-mediated increase in the activity of thermogenesis-promoting neurons in the DMH^1,2,9,11,32^. Thus, we postulate that, paralleling cold defense, the MnPO neurons whose activity is required for febrile BAT thermogenesis in rats are providing the glutamatergic excitation of BAT sympathoexcitatory neurons in the DMH which, in turn, drive BAT thermogenesis through excitation of BAT sympathetic premotor neurons in the rRPa.

A recent study proposes that lipopolysaccharide (LPS)-induced fever in mice occurs through PGE_2_-mediated inhibition of the EP3 receptor-expressing, POA glutamatergic neurons that project directly to hypothesized GABAergic interneurons in the rRPa^4^. Although this disinhibition-based model for febrile thermogenesis in mice may complement that proposed to mediate fever in rats^1,9,21^, neither model (disinhibition of rRPa premotor neurons in the mouse vs disinhibition of DMH thermogenesis-promoting neurons in rat) describes the source(s) of the excitation to the disinhibited neurons that would be necessary for their discharge to increase subsequent to their disinhibition, and thereby drive febrile thermogenesis. Although little is known about the central circuitry controlling thermoeffectors in mice, if, as in the rat, a glutamatergic excitatory input from the DMH is required for the febrile excitation of thermogenesis premotor neurons in the mouse rRPa, then an activation of DMH neurons would also be necessary for the expression of LPS-induced fever in mice. Therefore, we propose that a population of glutamatergic neurons in the MnPO provides the excitatory input to BAT sympathoexcitatory neurons in the DMH that is essential for the fever-induced increase in BAT thermogenesis in both rats and mice. It remains to be determined whether it is the same population of glutamatergic, DMH-projecting, BAT sympathoexcitatory neurons in MnPO that excites thermogenesis-promoting DMH neurons during both cold-evoked and PGE_2_-evoked BAT thermogenesis. The source(s) of the excitatory input that regulates the activity of these BAT sympathoexcitatory neurons in MnPO also remains to be identified.

In contrast to cool rats (Fig. 2), in warm rats, inhibition of neurons in the MPA did not increase BAT SNA (Fig. 4), consistent with a low level of activity in the MnPO glutamatergic input to DMH. The finding that subsequent blockade of GABA_A_ receptors in MnPO elicits a strong activation of BAT SNA (Fig. 4)^16^ suggests that a warm-active, GABAergic inhibition of the MnPO glutamatergic excitation of DMH neurons contributes to the low level of BAT SNA during skin/core warming. Warm-sensitive, GABAergic POA neurons^33^ are a potential source of this inhibitory regulation of BAT sympathoexcitatory neurons in MnPO (Fig. 8). Cutaneous warm receptor signaling via the dorsolateral LPB (LPBdl) may contribute to the discharge of these warm-sensitive, GABAergic neurons (Fig. 8), in addition to exciting the GABAergic neurons in the MPA that inhibit thermogenesis-promoting neurons in the DMH^34^. Thus, the cooling-evoked increase in the activity of BAT thermogenesis-promoting neurons in DMH may arise from a combination of their reduced inhibitory input from warm-excited, GABAergic neurons in MPA and an increased excitation from disinhibited glutamatergic neurons in MnPO (Fig. 8).

In conclusion, our study demonstrates the existence of a cooling-activated, glutamatergic projection from the MnPO to the DMH that provides an excitation of thermogenesis-promoting neurons in the DMH, which is essential for the activation of BAT thermogenesis during cold defense, and likely during fever as well. These results add new functional elements (Fig. 8) to the complex central thermoregulatory circuit. A better understanding of the central control of thermoregulation is essential for developing new therapeutic approaches for the rapid induction of hypothermia^35–40^, for the treatment of drug-resistant fever^3^, or for augmenting BAT thermogenic energy expenditure to improve glucose and energy homeostasis^2,3,41^.

## Materials and methods

### Animals

Male Wistar rats (300–400 g, Charles River Laboratories) were maintained in a standard 12 h/12 h light/dark cycle (lights on at 0900), with ad libitum access to standard chow and water. Experiments were performed in accordance with the Guide for the Care and Use of Laboratory Animals, 8^th^ edition (National Research Council, National Academies Press, 2010) and protocols were approved by the Institutional Animal Care and Use Committee of Oregon Health and Science University.

### Surgical and experimental procedures for BAT sympathetic nerve activity (SNA) recordings

As in our previous work^37,39^, rats initially anesthetized with isoflurane (3% in 100% O_2_) were transitioned to intravenous urethane (0.8 g/kg) and chloralose (80 mg/kg). A femoral arterial catheter was used for the acquisition of arterial pressure (AP) signal, from which heart rate (HR) was derived. Rats were positioned prone in a stereotaxic frame with the incisor bar at -4 mm below interaural zero.

A spinal clamp installed on the T_10_ vertebra was used to maintain the spine in a rigid and elevated position to create an optimal oil pool within which to record interscapular BAT SNA, and to reduce potential for respiratory-related artifacts in the BAT SNA recordings. Rats were paralyzed with D-tubocurarine (0.3 mg initial dose, 0.1 mg/hr supplements) and artificially ventilated (100% O_2_, 60–70 cycles/min, tidal volume 3–3.5 ml). Small adjustments in minute ventilation were made to maintain basal mixed-expired CO_2_ levels between 3 and 4.5%. Thermocouples (Physitemp with TC-2000 Thermocouple Meter Sable Systems International) were placed on the shaved abdominal skin to measure skin temperature (T_SKIN_), 6 cm into the rectum to measure T_CORE_, and into the medial aspect of the left interscapular BAT (iBAT) pad to measure BAT temperature (T_BAT_). A water-perfused thermal blanket was used to change T_SKIN_ and T_CORE_ according to the specific requirements of the experimental protocols. Perfusion with warm water stimulates warm thermoreceptors in the trunk skin, leading to inhibition of BAT SNA. Normally, T_CORE_ was maintained at ~ 37 °C by perfusing the water blanket with warm water. Perfusion of the thermal blanket with cool water stimulates cutaneous cold receptors in the trunk skin beneath the water blanket and elicits an elevated level of BAT SNA.

Postganglionic BAT SNA was recorded from the central cut end of a nerve bundle dissected from the ventral surface of the right iBAT pad after dividing the iBAT pad along the midline and reflecting it laterally. The BAT SNA signal, recorded with bipolar hook electrodes, was filtered (1–300 Hz) and amplified (20,000 × ; Cyberamp 380, Axon Instruments). The quality of the BAT sympathetic nerve recording was verified by evoking BAT nerve potentials with electrical stimulation of the sympathetic premotor neurons in the rRPa (relative to lambda: − 3.0 mm posterior, 0.0 mm lateral, − 9.2 mm ventral; incisor bar at − 4 mm).

### Drugs

All drugs were obtained from Tocris (Bio-Techne Corporation, Minneapolis, MN). The GABA_A_ receptor agonist muscimol (1 mM), the GABA_A_ receptor antagonist bicuculline (250 µM), N-methyl-D-aspartate (NMDA, 2 mM), the NMDA receptor antagonist (2R)-amino-5-phosphonopentanoate (AP5, 5 mM), and the AMPA/kainate receptor antagonist 6-cyano-7-nitroquinoxaline-2,3-dione disodium salt hydrate (CNQX, 5 mM) were dissolved in saline. The EP3 receptor agonist prostaglandin E2 (PGE_2_, 1 mg/mL) was dissolved in artificial cerebral spinal fluid (aCSF).

### Microinjection procedures

Intraparenchymal nanoinjections of drugs and tracers were accomplished via glass micropipettes (outer tip diameter: 20–30 µm) as described^37^. The coordinates for the brain intraparenchymal injections were adapted from a rat brain atlas^43^: MPA (0.5 mm caudal to bregma, 0.4 mm lateral to bregma, 7.5 mm ventral to the brain surface; incisor bar at − 4 mm); MnPO (0.0 mm caudal to bregma, 0.0 mm lateral to bregma, 6.5 mm ventral to the brain surface; incisor bar at − 4 mm); DMH (3.2 mm caudal to bregma, 0.4 mm lateral to bregma, − 7.5 mm ventral to the brain surface; incisor bar at − 4 mm). The microinjection sites were marked by pressure microinjection of fluorescent polystyrene microspheres (1:10 dilution of FluoroSpheres, F8797, F8801, or F8803, Invitrogen) to localize the injection sites postmortem^37^.

### Procedure for pre-DMH transection

The procedure for pre-DMH transections was identical to that employed previously^39^, except that the knife (15 mm long, 2 mm wide, and 0.1 mm thick) was lowered into the brain, on the left and right sides of the superior sagittal sinus, at two different depths (~ 8 mm and ~ 9 mm). Preliminary experiments indicated that a − 8 mm depth transection maintained the cold-evoked BAT SNA response, and thus served as a control for the surgical procedure involved in making these transections. At the second cut depth (− 9 mm), the blade traversed the region just rostral to the principal dorsoventral extent of the DMH. We intentionally avoided the complete transection depth of ~ 10 mm because is known to produce thermoregulatory inversion^39^.

### Neuroanatomical experimental procedures

Adult male Wistar rats (240–400 g) were anesthetized with 3% isoflurane in 100% O_2_ and cholera toxin subunit b (CTb) conjugated with Alexa-488 (1 mg/ml, 120 nl) was stereotaxically injected into the right DMH (bregma: − 3.2 mm caudal, 0.4 mm lateral; 7.5 mm ventral to the brain surface; incisor bar at − 4 mm:) and with FluoroGold (FG, 2%, 30 nl) into the rRPa (relative to lambda: − 3.0 mm caudal, 0.0 mm lateral, − 9.2 mm ventral to brain surface; incisor bar at − 4 mm). After each injection, the glass micropipette was left in place for 5 min. Rats were treated with intramuscular antibiotic (40,000 units/kg penicillin G) and analgesic (0.05 mg/kg buprenorphine) and subcutaneous saline (3 ml). One week after tracer injections, 4 rats were exposed to a cold ambient (T_AMB_: 10 °C) and 5 rats were exposed to a warm ambient (T_AMB_: 30 °C) for 2 h (2 h) to elicit c-Fos expression as an indicator of neuronal activation. To maximize c-Fos expression, rats were maintained for 24 h at a T_AMB_ of 30 °C prior to the 2 h cold exposure or maintained for 24 h at a T_AMB_ of 10 °C prior to the 2 h warm exposure.

Following the 2 h cold or warm exposure, rats were deeply anesthetized with pentobarbital (80 mg/kg i.p.) and transcardially perfused with saline followed by 4% paraformaldehyde (PFA). The brains were removed and post-fixed in 4% PFA for 2 h and equilibrated overnight in 20% sucrose in 10 mM sodium phosphate buffered saline (PBS; pH 7.4) with 0.01% sodium azide. Serial coronal Sects. (30 µm) were cut with a freezing-stage microtome, collected sequentially in 6 sets, and stored in cryoprotectant with 0.01% sodium azide at − 20 °C. Sections containing the preoptic area were pre-incubated for 3 h in an antibody dilution solution (ADS) containing PBS, 0.3% Triton-X 100, 0.25% carrageenan, 0.02% NaN_3_, and 1% normal donkey serum, and incubated overnight at room temperature in ADS containing the primary antibodies for c-Fos (rabbit anti-c-Fos , 1:10 K, Calbiochem) and for CTb (goat anti-CTb, 1:10 K, List Biologicals). After two washes in PBS containing 0.3% Triton-X 100 (TPBS, 20 mM), the sections were incubated for 1 h in ADS containing the secondary species-specific antibody for c-Fos (Alexa-594 donkey anti-rabbit, 1:500, Molecular Probes, cat #: A21207). After two washes in TPBS, the sections were incubated for 1 h in ADS containing the secondary antibody for CTb (Alexa-488 donkey anti-goat, 1:500, Molecular Probes, cat #: A11055). After incubation, the sections were washed in PBS and mounted on coated slides, air-dried and cover slipped with anti-fade mounting medium (Pro-Long Gold, Invitrogen).

To identify glutamatergic neurons in the MnPO that project to DMH (CTb-labeled) and are responsive to cold exposure, vesicular glutamate transporter 2 (VGluT2)- and c-fos-mRNA transcripts were detected by in situ hybridization using the RNAScope procedure (Advanced Cell Diagnostics, Hayward, CA), followed by immunofluorescence labeling of CTb. Briefly, sections containing the preoptic area were mounted on Superfrost Plus slides and dried at 4 °C overnight. The slides were post-fixed quickly in cold 4% PFA, dried, and subjected to the RNAscope multiplex fluorescent assay following manufacturer instructions. After VGluT2 and c-fos mRNA detection, the slides were blocked in 4% normal donkey serum in PBS for 1 h, followed by incubation in primary antisera containing goat anti-CTb (1:1 K) overnight at room temperature. Slides were washed in PBS and incubated for 2 h in the species-specific secondary antibody for CTb (Alexa-488 donkey anti-goat, 1:500, Jackson ImmunoResearch, cat #: 705–545-003). Slides were washed in PBS and coverslipped with anti-fade mounting medium (Pro-Long Gold, Invitrogen). VGluT2 mRNA transcripts were labeled with the Atto 550 nm probe and appeared as cytoplasmic punctate red fluorescence, whereas c-fos mRNA transcripts were labeled with the Atto 647 nm probe (far red) and were visualized as cytoplasmic punctate in blue (assigned color in the captured images). CTb-ir neurons displayed cytoplasmic green fluorescence.

### Tissue Analysis

The neuroanatomic designations of the injection sites in the rRPa and DMH and those of CTb-, FG-, c-Fos- and VGluT2-labeled neurons in the POA are based on the stereotaxic rat brain atlas of Paxinos and Watson^43^. Photomicrographs of brain sections and labeled neurons were obtained using image capturing software (Keyence BZ-H4XD) integrated with the Keyence BZ-X710 fluorescence microscope. RNAScope images were captured with SimplePCI software (C-Imaging Systems) in an Olympus BX-51 fluorescence microscope. The photomicrographs were assembled into plates using Adobe Photoshop to adjust contrast and brightness without altering the original colors.

### Data acquisition

Physiological variables were digitized (Micro 1401 MKII; Cambridge Electronic Design) at the following rates: BAT SNA (1 kHz), T_BAT_ (5 Hz), T_CORE_ (5 Hz), T_SKIN_ (5 Hz), T_PAW_ (5 Hz), expired CO_2_ (200 Hz), and AP (200 Hz), and recorded for subsequent analysis using Spike 2 software (Cambridge Electronic Design). A continuous measure (4 s bins) of BAT SNA amplitude was calculated as the root mean square (rms, square root of the total power in the 0.1 to 20 Hz band of the autospectrum) value of sequential 4 s segments of the BAT SNA signal.

### Data and statistical analysis

For analysis of physiological variables, the data were averaged into 30 s bins, and group data were reported as mean ± standard error of the mean (SEM). To account for slight differences in nerve recording, characteristic among experiments, raw BAT SNA values in individual experiments were expressed as a percentage of baseline value (% BL), where baseline is the lowest rms level of BAT SNA, recorded under warm conditions (T_CORE_ > 36 °C).

All statistical analyses were performed using Prism software (Version 6, GraphPad Software, Inc.). The statistical comparisons were performed using either Student t-test for which t value are reported, or repeated measure one-way ANOVA, for which F value and degrees of freedom (F_x,x_) are reported. Bonferroni post-hoc comparison was used for individual comparisons (IC) between a single control value taken before the treatment (i.e., 120 s pre-treatment) vs. a single value taken at the nadir or at the peak following treatment response (i.e., 600 s post-treatment). The modified t* value and p value for each IC is reported. Statistical results with *p* < 0.05 were considered significant.

## Supplementary information

Supplementary file1

## Data Availability

The data that support the findings of this study are available from the corresponding author upon reasonable request.

## References

[1] Nakamura K (2011). Central circuitries for body temperature regulation and fever. Am. J. Physiol. Regul. Integr. Comp. Physiol..

[2] Morrison SF, Madden CJ, Tupone D (2014). Central neural regulation of brown adipose tissue thermogenesis and energy expenditure. Cell Metab..

[3] Tupone D, Madden CJ, Morrison SF (2014). Autonomic regulation of brown adipose tissue thermogenesis in health and disease: potential clinical applications for altering BAT thermogenesis. Front. Neurosci..

[4] Machado NLS, Bandaru SS, Abbott SBG, Saper CB (2020). EP3R-expressing glutamatergic preoptic neurons mediate inflammatory fever. J. Neurosci..

[5] Nakamura K, Morrison SF (2011). Central efferent pathways for cold-defensive and febrile shivering. J. Physiol..

[6] Tan CL (2016). Warm-sensitive neurons that control body temperature. Cell.

[7] Tan CL, Knight ZA (2018). Regulation of body temperature by the nervous system. Neuron.

[8] Morrison SF, Nakamura K (2011). Central neural pathways for thermoregulation. Front. Biosci..

[9] Nakamura Y (2005). Direct pyrogenic input from prostaglandin EP3 receptor-expressing preoptic neurons to the dorsomedial hypothalamus. Eur. J. Neurosci..

[10] Conceicao EPS, Madden CJ, Morrison SF (2019). Neurons in the rat ventral lateral preoptic area are essential for the warm-evoked inhibition of brown adipose tissue and shivering thermogenesis. Acta Physiol. (Oxf.).

[11] Madden CJ, Morrison SF (2004). Excitatory amino acid receptors in the dorsomedial hypothalamus mediate prostaglandin-evoked thermogenesis in brown adipose tissue. Am. J. Physiol. Regul. Integr. Comp. Physiol..

[12] Nakamura Y, Nakamura K, Morrison SF (2009). Different populations of prostaglandin EP3 receptor-expressing preoptic neurons project to two fever-mediating sympathoexcitatory brain regions. Neuroscience.

[13] Osaka T (2008). Blockade of prostaglandin E2-induced thermogenesis by unilateral microinjection of GABAA receptor antagonist into the preoptic area. Brain Res..

[14] Hunt JL, Zaretsky DV, Sarkar S, Dimicco JA (2010). Dorsomedial hypothalamus mediates autonomic, neuroendocrine, and locomotor responses evoked from the medial preoptic area. Am. J. Physiol. Regul. Integr. Comput. Physiol..

[15] Morrison SF (2016). Central control of body temperature. F1000Res.

[16] Nakamura K, Morrison SF (2008). Preoptic mechanism for cold-defensive responses to skin cooling. J. Physiol..

[17] Yoshida K, Li X, Cano G, Lazarus M, Saper CB (2009). Parallel preoptic pathways for thermoregulation. J. Neurosci..

[18] Hermann DM, Luppi PH, Peyron C, Hinckel P, Jouvet M (1997). Afferent projections to the rat nuclei raphe magnus, raphe pallidus and reticularis gigantocellularis pars alpha demonstrated by iontophoretic application of choleratoxin (subunit b). J. Chem. Neuroanat..

[19] Cano G (2003). Anatomical substrates for the central control of sympathetic outflow to interscapular adipose tissue during cold exposure. J. Comp. Neurol..

[20] Nakamura K, Morrison SF (2007). Central efferent pathways mediating skin cooling-evoked sympathetic thermogenesis in brown adipose tissue. Am. J. Physiol. Regul. Integr. Comp. Physiol..

[21] Morrison SF, Nakamura K (2019). Central Mechanisms for Thermoregulation. Annu. Rev. Physiol..

[22] Kataoka N, Hioki H, Kaneko T, Nakamura K (2014). Psychological stress activates a dorsomedial hypothalamus-medullary raphe circuit driving brown adipose tissue thermogenesis and hyperthermia. Cell Metab..

[23] Machado NLS (2018). A glutamatergic hypothalamomedullary circuit mediates thermogenesis, but not heat conservation, during stress-induced hyperthermia. Curr. Biol..

[24] Wanner SP (2017). Cold-induced thermogenesis and inflammation-associated cold-seeking behavior are represented by different dorsomedial hypothalamic sites: a three-dimensional functional topography study in conscious rats. J. Neurosci..

[25] Zhao ZD (2017). A hypothalamic circuit that controls body temperature. Proc. Natl. Acad. Sci. U. S. A..

[26] Geerling JC (2016). Genetic identity of thermosensory relay neurons in the lateral parabrachial nucleus. Am. J. Physiol. Regul. Integr. Comp. Physiol..

[27] Nakamura K, Morrison SF (2008). A thermosensory pathway that controls body temperature. Nat. Neurosci..

[28] Tupone D, Madden CJ, Cano G, Morrison SF (2011). An orexinergic projection from perifornical hypothalamus to raphe pallidus increases rat brown adipose tissue thermogenesis. J. Neurosci..

[29] Blondin DP (2015). Contributions of white and brown adipose tissues and skeletal muscles to acute cold-induced metabolic responses in healthy men. J. Physiol..

[30] Nakamura K (2000). Immunohistochemical localization of prostaglandin EP3 receptor in the rat nervous system. J. Comp. Neurol..

[31] Lazarus M (2007). EP3 prostaglandin receptors in the median preoptic nucleus are critical for fever responses. Nat. Neurosci..

[32] Zaretskaia MV, Zaretsky DV, DiMicco JA (2003). Role of the dorsomedial hypothalamus in thermogenesis and tachycardia caused by microinjection of prostaglandin E2 into the preoptic area in anesthetized rats. Neurosci. Lett..

[33] Lundius EG, Sanchez-Alavez M, Ghochani Y, Klaus J, Tabarean IV (2010). Histamine influences body temperature by acting at H1 and H3 receptors on distinct populations of preoptic neurons. J. Neurosci..

[34] Nakamura, K. *Afferent Pathways for Autonomic and Shivering Thermoeffectors* Vol. 156 263–279 (Elsevier, Amsterdam, 2018). 10.1016/B978-0-444-63912-7.00016-3 30454594

[35] Drew KL, Romanovsky AA, Stephen TK, Tupone D, Williams RH (2015). Future approaches to therapeutic hypothermia: a symposium report. Temperature (Austin).

[36] Tupone D, Morrison S (2014). Hypothermia, torpor and the fundamental importance of understanding the central control of thermoregulation. Temperature (Austin).

[37] Tupone D, Madden CJ, Morrison SF (2013). Central activation of the A1 adenosine receptor (A1AR) induces a hypothermic, torpor-like state in the rat. J. Neurosci..

[38] Tupone D, Madden CJ, Morrison SF (2013). Highlights in basic autonomic neurosciences: central adenosine A1 receptor—the key to a hypometabolic state and therapeutic hypothermia?. Auton. Neurosci..

[39] Tupone D, Cano G, Morrison SF (2017). Thermoregulatory inversion: a novel thermoregulatory paradigm. Am. J. Physiol. Regul. Integr. Comp. Physiol..

[40] Cerri M (2013). The inhibition of neurons in the central nervous pathways for thermoregulatory cold defense induces a suspended animation state in the rat. J. Neurosci..

[41] Townsend KL (2017). Reestablishment of energy balance in a male mouse model with POMC neuron deletion of BMPR1A. Endocrinology.

[42] Nakamura K, Morrison SF (2010). A thermosensory pathway mediating heat-defense responses. Proc. Natl. Acad. Sci. U. S. A..

[43] Paxinos, G. & Watson, C. *The Rat Brain in Stereotaxic Coordinates* 6th edn 1–456 (Elsevier, 2007).

