# Supplementary material for: Median preoptic area neurons are required for the cooling and febrile activations of brown adipose tissue thermogenesis in rat
Source: Sci Rep. 2020 Oct 22;10:18072. doi: 10.1038/s41598-020-74272-w (PMC7581749; doi:10.1038/s41598-020-74272-w)
Supplement: Supplementary file 1 — Supplementary file1 [file 41598_2020_74272_MOESM1_ESM.pdf]

Supplementary Information

Median preoptic area neurons are required for the cooling and febrile activations of brown adipose tissue thermogenesis in rat.

Ellen Paula Santos da Conceição[1], Shaun F. Morrison[1], Georgina Cano[2], Pierfrancesco Chiavetta[1], and *Domenico Tupone[1, 3]

[1]Department of Neurological Surgery, Oregon Health & Science University, Portland, OR 97239, USA.

[2]Department of Neuroscience, University of Pittsburgh, Pittsburgh, PA 15260, USA.

[3]Department of Biomedical and Neuromotor Science, University of Bologna, Bologna 40126, Italy.

***Corresponding author:** domenico.tupone@gmail.com

S1. Saline injection in MnPO does not influence febrile BAT thermogenesis elicited by PGE_2 in MPA

aCSF injection in MnPO did not affect the PGE_2-evoked elevation in BAT SNA (Fig. 1A; pre-aCSF: 2270 ± 657.8% BL vs. post-aCSF: 2410 ± 472.9% BL, $F_{(40,3)}$ = 0.9088, p=0.6267; $IC_{(-120s\ vs\ 900s)}$, n = 4, t*=0.5728, p>0.05), the expired CO_2 (pre-aCSF: 4.0 ± 0.5% vs. post-aCSF: 4.2 ± 0.5%, $F_{(40,3)}$ = 2.85, p<0.0001; IC (-120s vs 900s), n = 4, t*=1.922, p>0.05) or HR (pre-aCSF: 493 ± 17.8 bpm vs. post-aCSF: 505 ± 17.9 bpm, $F_{(40,3)}$ = 1.430, p=0.0718; $IC_{(-120s\ vs\ 900s)}$, n = 4, t*=1.291, p>0.05). We observed a small, but significant, increase in T_{BAT} after the aCSF injection (pre-aCSF: 36.8 ±

0.7 °C vs. post-aCSF: 37.3 ± 0.8 °C, $F_{(40,3)}$ = 21.98, p<0.0001; $IC_{(-120s\ vs\ 900s)}$, n = 4, t*=8.396, p<0.05). This increase in T_{BAT} was not caused by the aCSF injection itself, but rather it was the continuation of the slowly rising increase (i.e., a delayed plateau) in T_{BAT} that was part of the response to the administration of PGE_2.

S2. Saline injection in MPA or MnPO did not influence cold-evoked increases in BAT SNA and BAT thermogenesis

Injection of saline vehicle into the MPA did not alter the level of cold-evoked BAT SNA (Fig. 3; pre-saline in MPA level of 1151 ± 486.4% BL vs. post-saline in MPA level of 1417 ± 485.8% BL, $F_{(40,4)}$ = 0.9360, p=0.5669; $IC_{(-120s\ vs\ 600s)}$, n = 5, t*=0.022, p>0.05). In contrast to rats treated with muscimol in MPA (Fig. 2), nanoinjection of saline vehicle in MPA did not affect the ability of skin warming (ΔT_{SKIN}: +5.6 ± 1.2 ºC from a baseline of 34.0 ± 0.6 ºC; $F_{(8,3)}$ = 17.26, p<0.0001; $IC_{(-120s\ vs\ 120s)}$, n = 4, t*= 6.5, p<0.05) to reduce the skin/core cooling-evoked increases in BAT SNA (Δ BAT SNA: pre-skin warming +1366 ± 352.9% BL vs. post-skin warming +799.1 ± 319.4% BL, $F_{(8,3)}$ = 3.781, p=0.0053; $IC_{(-120s\ vs\ 120s)}$, n = 4, t*=4.294, p<0.05). Nanoinjection of saline vehicle in the MnPO did not produce any change in the level of cold-evoked BAT SNA (Fig. 3A; pre-saline: 1243 ± 438.0% BL vs post-saline: 1159 ± 420.7% BL, $F_{(40,4)}$ = 0.4816, p=0.9960; $IC_{(-120s\ vs\ 600s)}$, n =

5, t*=0.5549, p>0.05). These data demonstrate that the effects of nanoinjecting muscimol in either the MPA or the MnPO do not arise from either the volume injected or from the vehicle.

S3. Distributions of cold- or warm-activated POA neurons that project to DMH or rRPa

In agreement with a previous study[12], we found numerous CTb-immunoreactive (-ir) and FG-ir neurons in both the MnPO and MPA regions (Fig. 7A). Following CTb injections in the right DMH, we found an ipsilateral predominance of CTb-ir neurons in the MPA, as well as in the MnPO at rostral levels (e.g., Fig. 7A, bregma 0.0 mm to -0.24 mm). Following FG injections in the rRPa, FG-ir neurons were distributed bilaterally in the MPA and MnPO (Fig. 7A). Consistent with the previous study[12], we observed double-labeled neurons (i.e., CTb-ir and FG-ir, indicating MnPO neurons with bifurcating axons projecting to both DMH and rRPa) in the MnPO (at bregma level: 23.3 ± 6.5, at -0.12 mm caudal to bregma level: 9.3 ± 1.8, and at -0.24 mm caudal to bregma level: 15.8 ± 3.7) and in the MPA (at -0.12 mm caudal to bregma level: 7.8 ± 2.7 and at -0.24 mm caudal to bregma level: 10.4 ± 3.4). Relative to the total number of CTb-ir neurons in the MnPO, there was only a small number of double-labeled CTb-ir and FG-ir neurons in the MnPO (at bregma level: 6.9 ± 1.8%, at -0.12 mm caudal to bregma level: 6.6 ± 1.5%, and at -0.24 mm caudal to bregma level: 8.7 ± 1.6%). Both cold and warm exposure induced c-Fos expression in neurons in the MnPO and MPA. In the MnPO, the number of c-Fos-ir neurons was significantly greater in cold-exposed rats than in warm-exposed rats at the level of bregma (570.5 ± 21.2 for cold vs. 255.0 ± 69.1 for warm, n = 11, t=3.917 p=0.0029) and at -0.12 mm caudal to bregma level (260.0 ± 64.1 for cold vs. 94.6 ± 11.6 for warm, n = 11, t=2.861 p=0.0121). In the MPA, the number of c-Fos-ir neurons was also significantly greater in cold-

exposed rats than in warm-exposed rats at -0.12 mm caudal to bregma level (179 ± 19.4 for cold vs. 86.4 ± 21.9 for warm; n = 11, t=3.065, p=0.0091).

S4. Distribution and quantification of triple-labeled (CTb-ir, FG-ir, and c-Fos-ir) neurons in POA

In the MnPO, the number of double-labeled (CTb-ir and FG-ir) neurons that were also c-Fos-ir was not different between cold- and warm-exposed rats at the level of bregma (3.5 ± 0.8 for cold vs. 1.8 ± 0.8 for warm, t=0.5580, p=0.2658), at -0.12 mm caudal to bregma level (2.5 ± 1.3 for cold vs. 0.4 ± 0.4 for warm, n = 11, t=1.584 p=0.068), and at -0.24 mm caudal to bregma level (1.9 ± 1.4 for cold vs. 0.8 ± 0.6 for warm, n = 11, t=0.7965 p=0.2259). In the MPA, the number of double-labeled CTb-FG-ir neurons that were also c-Fos-ir was not significantly different between cold- and warm-exposed rats at -0.12 mm caudal to bregma level (3.0 ± 1.5 for cold vs. 1.6 ± 0.9 for warm, n = 11, t=0.8401 p=0.2143), and at -0.24 mm caudal to bregma level (3.7 ± 2.3 for cold vs. 0.6 ± 0.6 for warm, n = 11, t=1.507 p=0.087).
